# Efficacy of Thrombin Solution Injection Combined with Rapid Biopsy-Side Down Position Technique in CT–guided Lung Biopsy: A Propensity Score Matching Analysis

**DOI:** 10.2174/0115734056342141250320084906

**Published:** 2025-04-09

**Authors:** Baijintao Sun, Bing Li, Chuan Zhang, Yan Liu, Qing Zhang

**Affiliations:** 1 Department of Radiology, Affiliated Hospital of North Sichuan Medical College, Nanchong, China

**Keywords:** Lung, Biopsy, Pneumothorax, Incidence, Emphysema

## Abstract

**Objective:**

The objective of this study is to investigate the effect of thrombin solution injection combined with the rapid biopsy-side down position technique on the incidence of pneumothorax in emphysema patients following computed tomography (CT)-guided lung biopsy based on propensity score matching.

**Materials & Methods:**

A retrospective study was conducted on emphysema patients who underwent CT-guided percutaneous lung biopsy between May 2022 and July 2023. Patients were divided into two groups based on the use of the rapid biopsy-side-down position technique. Propensity score matching was then applied to explore correlations.

**Results:**

A total of 212 patients were included in the study. Before propensity score matching, there were no significant differences between Groups A and B in terms of sex, lesion size, puncture path length, or patient positioning in multivariate logistic regression analysis. After matching with a 1:1 ratio, 41 patients were successfully paired. Logistic regression analysis revealed that the rapid biopsy-side down position technique was significantly correlated with a reduced incidence of pneumothorax (p = 0.027), serving as a protective factor.

**Conclusion:**

The combination of thrombin solution injection and the rapid biopsy-side down position technique significantly reduces the incidence of pneumothorax in emphysema patients following CT-guided lung biopsy.

## 1 INTRODUCTION

Computed tomography (CT)-guided lung biopsy offers high diagnostic accuracy, sensitivity, and specificity in detecting pulmonary lesions [1, 2]. However, complications such as pneumothorax, hemoptysis, hemorrhage, and pleuritic pain may occur post-procedure, with pneumothorax being the most common [3], followed by lung hemorrhage, which occurs in 13.7 - 16.9% of cases [4, 5, 6]. Emphysema is generally considered a relative contraindication for lung biopsy [4, 7, 8]. Therefore, minimizing the incidence of pneumothorax and hemorrhage after CT-guided lung biopsy in patients with emphysema, particularly those with concomitant lung nodules or unexplained masses, has become a key concern for both clinicians and patients.

Various strategies have been explored to reduce the incidence of pneumothorax after lung biopsy, including the use of tract sealants and patient positioning. Previous studies have shown that injecting thrombin solution into the biopsy tract can help reduce or prevent pneumothorax and hemorrhage. Thrombin promotes the conversion of fibrinogen into fibrin, forming a gelatinous fibrin plug that blocks small blood vessels and facilitates local hemostasis [9, 10]. Despite its potential benefits, international research on this approach remains limited. Additionally, patient positioning has also been investigated, with some studies suggesting that rapid patient rollover may decrease the incidence of pneumothorax by using gravity to seal the biopsy tract and prevent air leakage during CT-guided lung biopsy [3, 5, 11]. However, other studies have produced conflicting results, indicating that rapid patient positioning may not significantly impact the incidence of post-biopsy pneumothorax [12, 13]. Does combining thrombin solution injection with the rapid biopsy lateral positioning technique reduce the incidence of pneumothorax in patients with emphysema?

In our study, propensity score matching (PSM) analysis was employed to minimize the impact of confounding factors. The effectiveness of combining thrombin solution injection with the rapid biopsy-side down positioning technique after CT-guided lung biopsy in patients with emphysema was then evaluated. This approach is aimed at enhancing postoperative protection for patients and offering new insights for clinical treatment.

## 2 METHODS AND MATERIALS

### 2.1 Patients

Our research adhered to the principles outlined in the Declaration of Helsinki, and approval for the study was granted by the Ethics Board of the Affiliated Hospital of North Sichuan Medical College (2023ER043-1). All patients provided signed informed consent before the intervention and were informed to breathe normally during the procedure. A retrospective study was performed on the data acquired from participants who underwent CT-guided percutaneous lung biopsy at our institution between May 2022 and July 2023.

The inclusion criteria were patients who: (1) underwent CT-guided lung biopsy and had available CT images; (2) were aged ≥ 50 years old; (3) had emphysema; and (4) underwent needle tract injection with thrombin solution during CT-guided lung biopsy. The exclusion criteria were patients who (1) had repeated surgery (such as inadequate biopsy specimen from the lesion at pathological examination) and (2) experienced pneumothorax before the biopsy was performed.

**Fig. (1):**
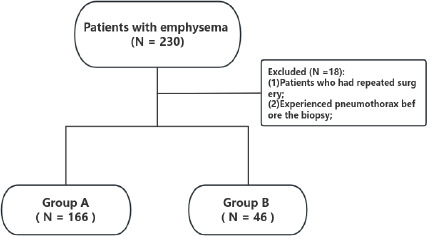
Flowchart of how this study population was selected and divided into two groups.

The patients were divided into two groups: Group A included the patients who received thrombin solution injection alone after lung biopsy, and Group B included the patients who received thrombin solution injection combined with rapid biopsy-side down position (Fig. **1**). Patient- (age and sex), lesion- (size and distance to the pleura), and technique-related (patient position, angle of the puncture needle, fissure puncture, and procedure duration) data were collected for each group. The distance to the pleura surface was defined as the distance along the biopsy needle path from the pleural puncture to the target lesion. The angle of the puncture needle was formed between the biopsy needle and the horizontal line. The fissure puncture was defined as the biopsy needle traversing the lung lobe fissure to the lesion (Table **1**).

### 2.2 Procedure

**Fig. (2):**
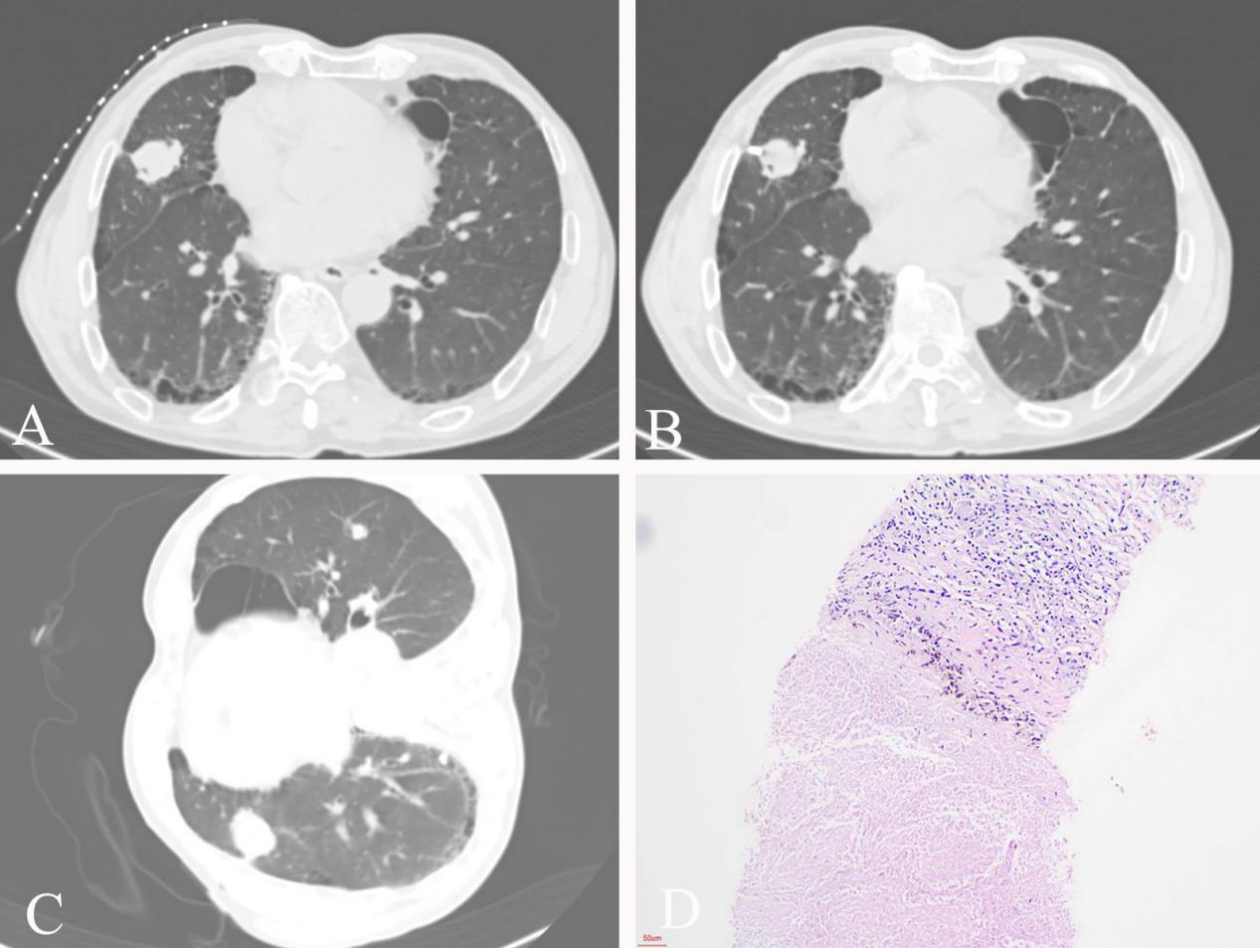
(**A**) An emphysema patient with a right lung middle lobe mass. (**B**) The tip of the needle in the tumor. (**C**) Rapid biopsy-side down position after biopsy and collected the CT images. (**D**) Pathological presentation of the specimens with fibrous tissue proliferation and coagulative necrosis.

**Fig. (3):**
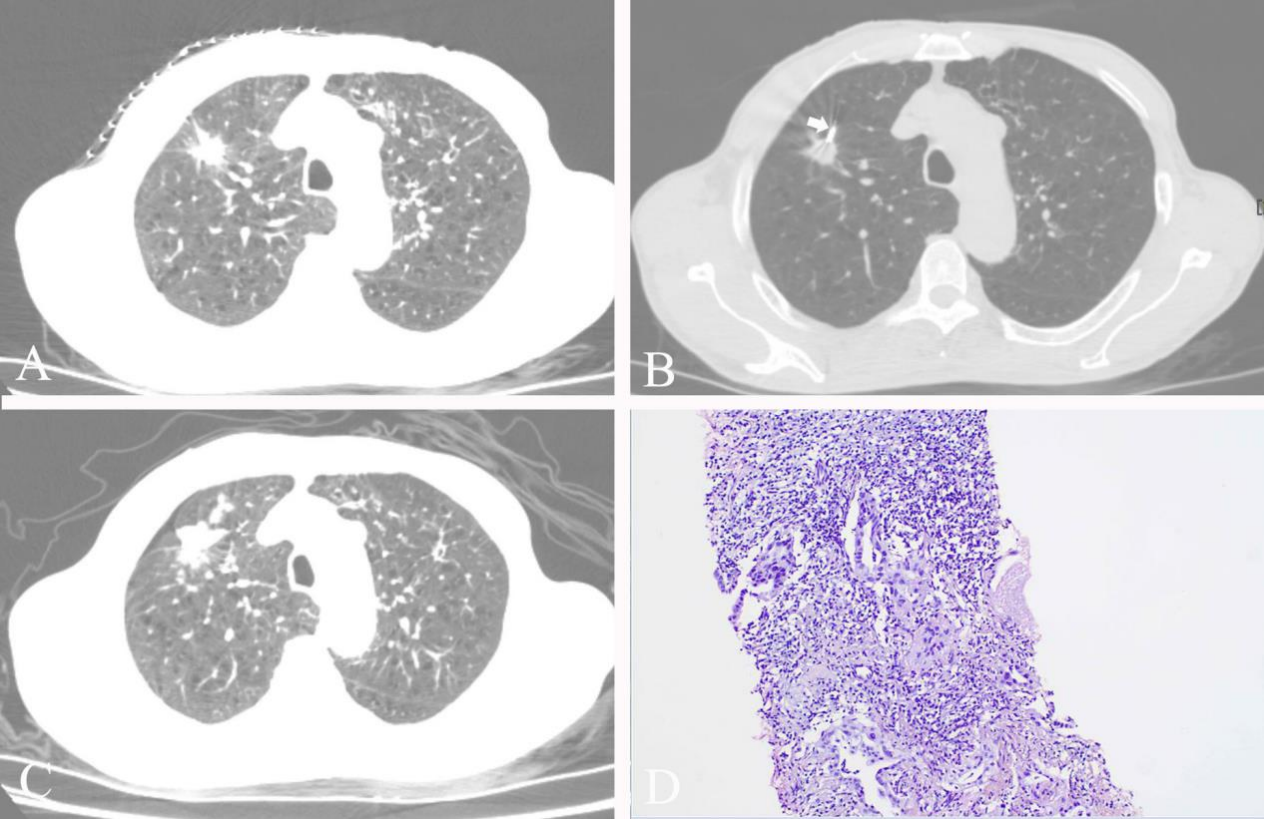
(**A**) An emphysema patient with a right lung upper lobe mass. (**B**) The tip of the needle in the tumor (white arrow). (**C**) the CT images proved pneumothorax occurrence. (**D**) Pathological presentation of the specimens was adenocarcinoma.

The procedure for both groups was performed under CT guidance (Royal Philips, MX16). Prior to the procedure, CT imaging of the entire lung (10-mm slice and 10-mm slice gap) was performed to identify the appropriate biopsy level. Next, a CT thin-layer scan with 3–5 mm of scanning thickness was used to determine the appropriate biopsy technique. The operator performed procedural CT acquisitions to ensure that the needle tip reached the target lesion properly. After needle removal, all of the patients were injected with thrombin solution (thrombin 0.5 ku with 10 ml normal saline) into the biopsy tract. Group A patients maintained their original positions, whereas group B patients underwent rapid biopsy-side-down position. Both groups subsequently underwent CT imaging of the entire lung to assess the presence of pneumothorax (Figs. **2** and **3**).

All of the procedures in this study were performed by the same interventional radiologist with 10 years of experience. Preoperative and postoperative CT images were independently reviewed by a senior radiologist and a junior radiologist to evaluate emphysema and pneumothorax. Any discrepancies between the two radiologists were resolved by consensus.

### 2.3 Propensity Score Matching

PSM analysis was conducted using the IBM SPSS Statistics (version 26.0). PSM was used to balance confounders and reduce potential selection bias between our groups [14, 15]. Logistic regression analysis was used to estimate propensity scores for each patient. A total of 8 relevant factors mentioned above were included in the regression model(including age, sex, lesion size, patient position, angle of puncture needle, puncture path length, time of procedure, and number of fissure punctures). PSM seeks to create 2 similar groups by matching them on a range of covariates.

In this study, 1 patient in group A was matched to 1 patient in group B with a 0.05 caliper level. If the matching procedure was successful, there would be no differences in the covariates between the two groups, achieving covariate balance.

### 2.4 Statistical Methods

The continuous variables were expressed as means and standard deviations (SD), and the categorical variables were expressed as numbers and frequencies (%). Continuous variables were evaluated with *t*-tests; chi-square tests were used for categorical variables.

Univariate and multivariate logistic regression analysis was used to identify factors related to pneumothorax incidence. The odds ratio (OR) and 95% confidence interval (95% CI) were calculated when appropriate (Table **2**). All of the data analyses were performed using IBM SPSS Statistics (version 26.0). Results were considered to be significant at *p* < 0.05.

## 3 RESULTS

A total of 212 patients with emphysema were divided into two groups: Group A had 166 patients (145 men; 87.3%), and Group B had 66 patients (33 men; 71.7%). In the univariate analysis, sex (*p* = 0.011), lesion size (*p* = 0.003), length of the puncture path (*p* < 0.001), patient position (*p* < 0.001), and pneumothorax(*p* < 0.037) showed statistically significant differences between Groups A and B. There were no statistically significant differences in age (*p* = 0.981), angle of the puncture needle (*p* = 0.183), duration of the procedure (*p* = 0.173), or the number of fissure punctures (*p* = 0.439) between the two groups (Table **1**).

However, in a multiple regression analysis of pneumothorax, there were no statistically significant differences in sex (*p* = 0.814), lesion size (*p* = 0.150), length of the puncture path (*p* = 0.532) and patient position (*p* = 0.094) (Table **2**).

There were eighty-two patients (41 pairs) that were successfully removed with 1:1 matching of the PSM results. After PSM analysis, no statistically significant differences were found between the two groups for sex, lesion size, puncture path length, patient position, age, angle of the puncture needle, procedure duration, or the number of fissure punctures (Table **3**). Logistic regression analysis showed that rapid biopsy-side down position after CT-guided lung biopsy was statistically significant as a protective factor against pneumothorax (*p* = 0.027; OR = 0.297, 95% CI = 0.101–0.870) (Table **4**).

## 4 DISCUSSION

In this study, PSM analysis was used to evaluate the impact of thrombin solution injection combined with the rapid biopsy-side down position technique on the incidence of pneumothorax in emphysema patients undergoing CT-guided lung biopsy. The primary finding was that the combination of thrombin solution injection and the rapid biopsy-side down positioning technique significantly reduced the incidence of pneumothorax in patients with emphysema. Both univariate and multiple regression analyses further confirmed that other factors did not contribute to the occurrence of pneumothorax in our study. In conclusion, these findings may offer a novel postoperative protection strategy for emphysema patients undergoing CT-guided percutaneous lung biopsy.

### 4.1 Analysis of Potential Clinical Confounders Affecting the Incidence of Pneumothorax

Pneumothorax is the most common complication following CT-guided lung biopsy, occurring in 12–45% of procedures [3, 14, 16, 17]. In patients with emphysema, the increased alveolar pressure heightens the risk of pneumothorax when some factors disturb the integrity of the lung parenchyma. Therefore, emphysema is a significant risk factor for pneumothorax development [18, 19]. In our study, univariate analysis revealed statistically significant differences between groups in terms of sex, lesion size, puncture path length, and patient position. These findings align with those of previous studies [1, 4, 18, 20, 21]. However, imbalances in potential confounding variables may skew the relationship between experimental factors and outcomes. To address this, these variables were included in a multiple regression analysis and found that none of them significantly influenced the incidence of pneumothorax in our study.

### 4.2 Thrombin Solution is an Effective Biopsy Tract Embolization Material for Lung Biopsy

Several studies have explored various sealant materials that can be injected into the biopsy tract to reduce or prevent pneumothorax, including autologous blood, gelatin sponge slurry, fibrin glue, normal saline, thrombin solution, and hydrogel plugs [22, 23]. Thrombin acts by converting fibrinogen into fibrin, which, in combination with coagulation factors, forms a gelatinous fibrin clot that blocks small blood vessels, thus achieving local hemostasis [9, 10]. Chen *et al*. [9] and Zheng *et al*. [10] reported that in elderly patients, the use of thrombin following biopsy effectively reduced the incidence of pneumothorax and facilitated local hemostasis. In our study, all patients received an injection of thrombin solution along the biopsy tract. It not only embolizes the biopsy tract to reduce the incidence of pneumothorax but also effectively achieves local hemostasis. It also suggests that the thrombin solution injection technique is a protective factor for CT-guided percutaneous lung puncture biopsy in patients.

### 4.3 Thrombin Solution Injection Combined with the Rapid Biopsy-side down Position Technique is a Protective Factor following Lung Biopsy

The biopsy-side-down position technique leverages gravity to seal the biopsy tract and prevent air leaks during CT-guided lung biopsy [2]. Zidulka *et al*. [24] demonstrated a reduction in the pneumothorax rate when dogs were positioned in the lateral decubitus position with the biopsy side down. Similarly, Kolu *et al*. [5] reported that maintaining the biopsy-side-down position after needle withdrawal could reduce the incidence of pneumothorax. O'Neill *et al*. [25] and Kim *et al*. [26] found that a rapid rollover to the biopsy-side-down position significantly decreased the rate of pneumothorax and the need for chest tube placement. These findings suggest that the biopsy-side-down position technique, particularly the rapid position technique, is effective in reducing the incidence of post-biopsy pneumothorax. Biopsies are commonly used in clinical practice. Hashimoto *et al*. demonstrated that biopsies are valuable for diagnosing bone and soft tissue metastases and identifying their primary site [27]. Although bone and soft tissue tumor punctures, as well as lung tumor punctures, differ in their puncture sites and potential complications, both procedures demand a high level of precision and careful postoperative monitoring. By leveraging the experience gained from puncturing tumors at various sites, the diagnostic and treatment process can be optimized, the success rate of the procedures can be enhanced, and the risks to patients can be minimized. Unlike conventional mass biopsies, lung biopsies pose a unique challenge due to the presence of air, which can result in pneumothorax—potentially life-threatening in severe cases. Therefore, there is an urgent need to develop more effective methods to reduce the incidence of pneumothorax. Further PSM analysis was employed to minimize bias, resulting in 41 successfully matched pairs (1:1) to eliminate potential confounding factors, including sex, lesion size, puncture path length, patient position, age, angle of the puncture needle, procedure duration, and the number of fissure punctures. This ensured that any remaining differences could be attributed to experimental factors. In this study, we combined the thrombin solution injection technique with the rapid biopsy-side down position technique and found that the incidence of pneumothorax was lower in emphysema patients who received the combined technique compared to those who did not undergo the rapid biopsy-side down position. The final results indicate that the combination of thrombin solution injection and rapid biopsy-side down positioning serves as a protective factor following lung biopsy in patients with emphysema.

## CONCLUSION

In our study, the combination of thrombin solution injection and the rapid biopsy-side down position technique following CT-guided lung biopsy significantly reduced the incidence of pneumothorax in patients with emphysema. The future integration of this technique into routine clinical practice for patients undergoing lung biopsy is warranted, as it has the potential to improve postoperative recovery and enhance patients' quality of life. These findings will be further validated through a prospective randomized study to confirm the efficacy and safety of this approach in diverse patient populations.

## LIMITATION AND FUTURE

Our study has several limitations. First, it employed a retrospective design, and the sample size was relatively small. Second, while propensity score matching (PSM) analysis effectively balanced baseline characteristics between the groups, it may have led to the loss of some observed values, potentially reducing the representativeness of the sample. Therefore, the accuracy of the results can be enhanced by increasing the sample size, conducting prospective randomized studies, and refining clinical data in future research.

## AUTHORS’ CONTRIBUTIONS

It is hereby acknowledged that all authors have accepted responsibility for the manuscript's content and consented to its submission. They have meticulously reviewed all results and unanimously approved the final version of the manuscript.

## Figures and Tables

**Table 1:** Characteristics of 212 patients before propensity score matching analysis.

**Variable**	**Group A (n = 166)**	**Group B (n = 46)**	***p*-valve**
Age (years; mean±SD)	68.12 ± 7.620	68.15 ± 9.482	0.981
Male sex n.(%)	145 (87.3)	33 (71.7)	0.011*
Lesion size (mm^2^; mean±SD)	1655.84 ± 1738.450	864.02 ± 761.214	0.003*
Patient position n.(%)			<0.001**
Supine	88 (53)	11 (23.9)	
Prone	78 (47)	35 (76.1)	
Angle of puncture needle(°; mean±SD)	58.19 ± 23.167	63.39 ± 23.243	0.183
Puncture path length(mm; mean±SD)	14.49 ± 13.759	24.80 ± 14.211	<0.001**
Procedure duration(min; mean±SD)	18.54 ± 6.428	17.11 ± 5.782	0.173
Fissure puncture n.(%)	2 (1.2)	2 (4.3)	0.439
Pneumothorax, n (%)	51 (30.7)	7(15.2)	0.037*

*p <0.05, **p < 0.001.

**Table 2:** Multivariate regression analyses of group A and B on influence factors for pneumothorax.

**Variable**	**OR**	**95% CI**	***p*-Value**
**Gender (male/female)**	0.907	0.402-2.046	0.814
**Lesion size**	1	1	0.150
**Patient position**	1.225	0.648-2.314	0.532
**Puncture path length**	1.02	0.997-1.043	0.094

*p <0.05, **p < 0.001

**Table 3:** Characteristics of 212 patients after propensity score matching analysis.

**Variable**	**Group A (n =41)**	**Group B (n = 41)**	***p*-valve**
**Age(years; mean ± SD)**	69.02 ± 6.721	68.07 ± 9.593	0.605
**Male sex n.(%)**	31 (75.6)	29 (70.7)	0.618
**Lesion size (mm^2^; mean±SD)**	935.80 ± 882.404	869.73 ± 777.470	0.720
**Patient position n.(%)**			1
Supine	11 (26.8)	11 (26.8)	
Prone	30 (73.2)	30 (73.2)	
**Angle of puncture needle (°; mean±SD)**	63.15 ± 22.080	62.66 ± 24.144	0.924
**Puncture path length(mm; mean±SD)**	23.24 ± 13.544	22.22 ± 11.319	0.711
**Procedure duration (min; mean±SD)**	17.78 ± 5.013	16.9 8± 6.002	0.512
**Fissure puncture n.(%)**	1 (2.4)	1 (2.4)	1

*p <0.05, **p < 0.001

**Table 4:** Logistic Regression analysis of factor influencing the occurrence of pneumothorax.

	β	SE	Wald	OR value	95%CI	*p*-value
Variable	-1.214	0.548	4.903	0.297	0.101-0.870	0.027
Constant	-0.550	0.324	2.878	0.577	/	0.090

Variable: Rapid biopsy-side down position

## Data Availability

The data and supportive information are available within the article.

## LIST OF ABBREVIATIONS

PSM: Propensity score matching
CT: Computed tomography

## STANDARDS OF REPORTING

STROBE guidelines were followed.

## References

[1] Grange R., Sarkissian R., Bayle-Bleuez S., Tissot C., Tiffet O., Barral F.G., Flaus A., Grange S. (2022). Preventive tract embolization with gelatin sponge slurry is safe and considerably reduces pneumothorax after CT-guided lung biopsy with use of large 16–18 coaxial needles.. Br. J. Radiol..

[2] Khorochkov E., Garvin G.J., Potoczny S., Kozak R.I. (2018). Injection of saline into the biopsy tract and rapid patient rollover decreases pneumothorax size following computed tomography–guided transthoracic needle biopsy.. Can. Assoc. Radiol. J..

[3] Heerink W.J., de Bock G.H., de Jonge G.J., Groen H.J.M., Vliegenthart R., Oudkerk M. (2017). Complication rates of CT-guided transthoracic lung biopsy: Meta-analysis.. Eur. Radiol..

[4] Lim W.H., Park C.M., Yoon S.H., Lim H.J., Hwang E.J., Lee J.H., Goo J.M. (2018). Time-dependent analysis of incidence, risk factors and clinical significance of pneumothorax after percutaneous lung biopsy.. Eur. Radiol..

[5] Kolu M., Yildirim I.O. (2020). Evaluation of risk factors in pneumothorax development after computerized tomography-guided transthoracic biopsy and management of complications.. Niger. J. Clin. Pract..

[6] Kim C.R., Sari M.A., Grimaldi E., VanderLaan P.A., Brook A., Brook O.R. (2024). CT-guided coaxial lung biopsy: Number of cores and association with complications.. Radiology.

[7] Guo Z., Shi H., Li W., Lin D., Wang C., Liu C., Yuan M., Wu X., Xiong B., He X., Duan F., Han J., Yang X., Yu H., Si T., Xu L., Xing W., Jinhua H., Wang Y., Xie H., Cui L., Gao W., He D., Liu C., Liu Z., Ma C., Pan J., Shao H., Tu Q., Yong L., Xu Y., Weihao Z., Qiang Z., Wang S. (2018). Chinese multidisciplinary expert consensus: Guidelines on percutaneous transthoracic needle biopsy.. Thorac. Cancer.

[8] Yoon S.H., Lee S.M., Park C.H., Lee J.H., Kim H., Chae K.J., Jin K.N., Lee K.H., Kim J.I., Hong J.H., Hwang E.J., Kim H., Suh Y.J., Park S., Park Y.S., Kim D.W., Choi M., Park C.M. (2021). 2020 Clinical practice guideline for percutaneous transthoracic needle biopsy of pulmonary lesions: A consensus statement and recommendations of the korean society of thoracic radiology.. Korean J. Radiol..

[9] Chen F.T., Zhong F.K., Bai Q.H., Zhu J., Yang Y., Cao L.F. (2018). Application of thrombin blocking technique in percutaneous lung biopsy in the elderly.. Chin J Geriatrics Res..

[10] Zheng H.J., Yang H.F., Du Y., Xu X.X., Li Y., Chen Y.K. (2011). Comparison of preventing common complications in percutaneous needle biopsy of lung using normal saline or thrombin solutions injection.. Radiol. Prat..

[11] Zeng L-C., Du Y., Yang H-F., Xie M-G., Liao H-Q., Zhang Y-D., Li L., Wang Q., Hu L., Xu X-X. (2015). Efficacy of an opposite position aspiration on resolution of pneumothorax following CT-guided lung biopsy.. Br. J. Radiol..

[12] Manhire A., Charig M., Clelland C., Gleeson F., Miller R., Moss H., Pointon K., Richardson C., Sawicka E. (2003). Guidelines for radiologically guided lung biopsy.. Thorax.

[13] Collings C.L., Westcott J.L., Banson N.L., Lange R.C. (1999). Pneumothorax and dependent versus nondependent patient position after needle biopsy of the lung.. Radiology.

[14] An W., Zhang H., Wang B., Zhong F., Wang S., Liao M. (2022). Comparison of CT-guided core needle biopsy in pulmonary ground-glass and solid nodules based on propensity score matching analysis.. Technol. Cancer Res. Treat..

[15] Kane L.T., Fang T., Galetta M.S., Goyal D.K.C., Nicholson K.J., Kepler C.K., Vaccaro A.R., Schroeder G.D. (2020). Propensity score matching.. Clin. Spine Surg..

[16] Wiener R.S., Schwartz L.M., Woloshin S., Welch H.G. (2011). Population-based risk for complications after transthoracic needle lung biopsy of a pulmonary nodule: An analysis of discharge records.. Ann. Intern. Med..

[17] He C., Zhao L., Yu H.L., Zhao W., Li D., Li G.D., Wang H., Huo B., Huang Q.M., Liang B.W., Ding R., Wang Z., Liu C., Deng L.Y., Xiong J.R., Huang X.Q. (2024). Pneumothorax after percutaneous CT-guided lung nodule biopsy: A prospective, multicenter study.. Quant. Imaging Med. Surg..

[18] Hiraki T., Mimura H., Gobara H., Iguchi T., Fujiwara H., Sakurai J., Matsui Y., Inoue D., Toyooka S., Sano Y., Kanazawa S. (2009). CT fluoroscopy-guided biopsy of 1,000 pulmonary lesions performed with 20-gauge coaxial cutting needles: Diagnostic yield and risk factors for diagnostic failure.. Chest.

[19] Kazerooni E.A., Lim F.T., Mikhail A., Martinez F.J. (1996). Risk of pneumothorax in CT-guided transthoracic needle aspiration biopsy of the lung.. Radiology.

[20] Sarajlic V., Vesnic S., Udovicic - Gagula D., Kuric H., Akhan O. (2021). Diagnostic accuracy and complication rates of percutaneous CT-guided coaxial needle biopsy of pulmonary lesions.. Diagn. Interv. Radiol..

[21] Renier H., Gérard L., Lamborelle P., Cousin F. (2020). Efficacy of the tract embolization technique with gelatin sponge slurry to reduce pneumothorax and chest tube placement after percutaneous CT-guided lung biopsy.. Cardiovasc. Intervent. Radiol..

[22] Babu S.B., Srinivasan S., Chung R., Chawla A., Tan H.K., Lohan R. (2020). Tract sealing with normal saline after percutaneous transthoracic lung biopsies.. J. Med. Imaging Radiat. Oncol..

[23] Maybody M., Muallem N., Brown K.T., Moskowitz C.S., Hsu M., Zenobi C.L., Jihad M., Getrajdman G.I., Sofocleous C.T., Erinjeri J.P., Covey A.M., Brody L.A., Yarmohammadi H., Deipolyi A.R., Bryce Y., Alago W., Siegelbaum R.H., Durack J.C., Gonzalez-Aguirre A.J., Ziv E., Boas F.E., Solomon S.B. (2019). Autologous blood patch injection versus hydrogel plug in CT-guided lung biopsy: A prospective randomized trial.. Radiology.

[24] Zidulka A. (1987). Position may reduce or stop pneumothorax formation in dogs receiving mechanical ventilation.. Clin. Invest. Med..

[25] O'Neill AC, McCarthy C, Ridge CA, Mitchell P, Hanrahan E, Butler M, Keane MP, Dodd JD (2012). Rapid needle-out patient-rollover time after percutaneous CT-guided transthoracic biopsy of lung nodules: Effect on pneumothorax rate.. Radiology.

[26] Kim J.I., Park C.M., Lee S.M., Goo J.M. (2015). Rapid needle-out patient-rollover approach after cone beam CT-guided lung biopsy: Effect on pneumothorax rate in 1,191 consecutive patients.. Eur. Radiol..

[27] Hashimoto K., Nishimura S., Ito T., Oka N., Akagi M. (2021). Limitations and usefulness of biopsy techniques for the diagnosis of metastatic bone and soft tissue tumors.. Ann. Med. Surg..

